# Progress of Phototherapy Applications in the Treatment of Bone Cancer

**DOI:** 10.3390/ijms222111354

**Published:** 2021-10-21

**Authors:** Jiachen Sun, Fei Xing, Joy Braun, Frank Traub, Pol Maria Rommens, Zhou Xiang, Ulrike Ritz

**Affiliations:** 1 Biomatics Group, Department of Orthopaedics and Traumatology, University Medical Center of the Johannes Gutenberg University, Langenbeckstr. 1, 55131 Mainz, Germany; sunjic123@126.com (J.S.); joybraun@uni-mainz.de (J.B.); frank.traub@unimedizin-mainz.de (F.T.); pol.rommens@unimedizin-mainz.de (P.M.R.); 2 Department of Orthopaedics, West China Hospital, Sichuan University, No. 37 Guoxue Lane, Chengdu 610041, China; xingfeihuaxi@163.com

**Keywords:** phototherapy, photodynamic therapy, photothermal therapy, bone cancer, tumor therapy, nanoparticles

## Abstract

Bone cancer including primary bone cancer and metastatic bone cancer, remains a challenge claiming millions of lives and affecting the life quality of survivors. Conventional treatments of bone cancer include wide surgical resection, radiotherapy, and chemotherapy. However, some bone cancer cells may remain or recur in the local area after resection, some are highly resistant to chemotherapy, and some are insensitive to radiotherapy. Phototherapy (PT) including photodynamic therapy (PDT) and photothermal therapy (PTT), is a clinically approved, minimally invasive, and highly selective treatment, and has been widely reported for cancer therapy. Under the irradiation of light of a specific wavelength, the photosensitizer (PS) in PDT can cause the increase of intracellular ROS and the photothermal agent (PTA) in PTT can induce photothermal conversion, leading to the tumoricidal effects. In this review, the progress of PT applications in the treatment of bone cancer has been outlined and summarized, and some envisioned challenges and future perspectives have been mentioned. This review provides the current state of the art regarding PDT and PTT in bone cancer and inspiration for future studies on PT.

## 1. Introduction

Bone cancer is divided into primary bone cancer and metastatic bone cancer, depending on whether the tumors invading the bone tissue are primary tumors or metastatic tumors. Primary malignant bone tumors include osteosarcoma, chondrosarcoma, and Ewing’s sarcoma, among others, which often occur in children and adolescents, accounting for about 6% of all cancers [1,2]. Among them, osteosarcoma is the second leading cause of tumor-related deaths in adolescents [3]. The early symptoms of primary bone cancer are not obvious, and patients often have pathological fractures or severe pain before going to the doctor. However, the invasion of primary malignant bone tumor progresses rapidly and can metastasize to other organs, especially the lung, so the early diagnosis and the treatment of primary bone cancer is difficult [4,5,6,7]. Bone metastases often occur in breast cancer, prostate cancer, lung cancer, liver cancer, kidney cancer, and so on. 65–80% of patients with breast cancer and prostate cancer develop bone metastases [8,9,10,11]. Metastatic bone cancer usually occurs in the spine and pelvis, accompanied by corresponding motor dysfunction and neurological symptoms of the affected tissue, as well as pathological fractures, pain, and other symptoms [12,13]. At present, the clinical treatment of bone cancer includes wide surgical resection, radiotherapy, and chemotherapy, often used in combination [14,15]. However, some tumor cells may remain in the local area after resection, and some bone tumors are insensitive to radiotherapy and have a tendency to be resistant to chemotherapy, leading to postoperative recurrence and metastasis [16,17]. In addition, the limb dysfunction caused by surgery and the damage to other physiological cells and tissues caused by radiotherapy or chemotherapy, have also seriously affected the life quality and mental health of patients [18,19]. Therefore, the treatment of bone cancer and other malignant tumors requires alternatives with an efficient and safe strategy.

Phototherapy (PT) involves the local exposure of patients to light to treat disease, including photodynamic therapy (PDT) and photothermal therapy (PTT). Both these therapies have been widely studied for cancer treatments in recent years, as they can eliminate tumor cells without damaging normal tissues [20,21]. PDT is a minimally invasive technique of treating tumor disease with photosensitizer (PS) and light activation. The PS that selectively accumulates in the tumor tissue can be activated by light of a specific non-thermal wavelength to produce reactive oxygen species (ROS), known as singlet oxygen, which can oxidize with nearby biological macromolecules in the tumor cells and thus cause cytotoxicity and cell death [22,23,24]. PTT is also a minimally invasive and highly efficient antitumor approach, which is based on photothermal agent (PTA) with high photothermal conversion efficiency [25,26]. The PTA can gather near the tumor tissue using targeted recognition technology and convert light energy into heat energy to kill cancer cells, as the cancer cells are more sensitive to high temperature than normal cells [27,28,29]. Furthermore, both PDT and PTT can be combined with other treatment methods to ablate tumors synergistically [30,31,32,33]. Given the difficulty of treating bone cancer and the broad prospects for PT, it is imperative to analyze and summarize the application progress of PT for bone cancer in the past three decades and present some envisioned challenges and future perspectives.

## 2. PDT

PDT was first discovered to damage paramecium cultured in a fluorescent dye, and then Dougherty et al. developed a variety of available PSs and excitation light sources, and applied them in the field of oncology in the 1970s [34,35]. At present, PDT has been proven to have ideal therapeutic effects of cancers, bacterial infections, skin diseases, and so on [36,37,38]. PDT has three crucial elements including PS, light source, and oxygen [39,40]. The anti-tumor effect of PDT is achieved by inducing direct cytotoxic effects on cancer cells (apoptosis, necrosis, and/or autophagy), destroying the tumor vasculature, and causing local inflammation followed by the systemic immunity [41]. PS can be selectively taken up by tumor tissues and can accumulate in tumor cells, while normal tissues take up less or rapidly metabolize the drug [42,43]. After uptake, the local tumor tissue is irradiated with light of a specific wavelength, and the nontoxic PS is activated to produce a large amount of highly reactive singlet oxygen, which causes the aforementioned biological responses of tumor cells and tissues. Finally, the growth of tumor is inhibited or tumor cells are ablated. In addition, the surrounding normal cells are protected from the PDT-induced cytotoxicity, because physiological cells in the tumor surrounding tissue are less sensitive to the toxicity of ROS [44,45,46]. Therefore, PDT has become an efficient, safe, convenient, and affordable strategy for tumor treatment.

Since the 1980s, hundreds of PSs have been studied, and some have been used in clinical trials [47]. There are currently three generations of PSs [48]. Most of the PSs used in tumor therapy are porphyrins, based on a tetrapyrrole structure which is similar to that of the protoporphyrin contained in hemoglobin [41]. Hematoporphyrin derivative (HpD), the most used first-generation PS which later became known as Photofrin, has been applied for the treatment of lung cancer, bladder cancer, esophageal cancer, and early stage cervical cancer [49]. However, the maximum absorption of HpD is at ~630 nm, leading to poor tissue penetration. In addition, the lack of specificity and the cutaneous phototoxicity also limit the widespread use of HpD, stimulating the development of new PSs [50,51,52]. The second-generation PSs include aminolevulinic acid (ALA), benzoporphyrin derivatives (BPDs), acridine orange (AO), and chlorins, among others. They have near infrared (NIR) absorption and high singlet oxygen quantum yield, and thus are characterized by higher efficiency and better penetration to deeply located tissues [53,54,55]. The third-generation PSs generally refer to the modifications of the first- and second-generations based on the synthesis of substances with higher affinity to the tumor tissue [56,57]. The applications of targeted recognition technology and nanocarriers have further improved the selectivity and safety of PS, and are conducive to the combination with other treatment methods such as chemotherapy, radiotherapy, and immunotherapy [58,59,60]. Both of the second- and third-generation PSs are the main directions of current studies.

The light source is another significant component of PDT. Each PS needs a corresponding appropriate light source. At present, light sources include the xenon lamp, light emitting diode (LED), laser beam, and fiber optic devices [61,62,63]. Some scholars believe the use of wavelengths between 600–850 nm is optimal for PDT which is called therapeutic window, while others think the region between 600 and 1200 nm is appropriate for PDT and can be called the optical window of tissue. However, the light with an absorption wavelength exceeding 800 nm will not have enough energy to induce a photodynamic reaction [41,49]. To improve the penetration capacity of light the light sources can be placed near the deep tissue via minimally invasive surgeries such as endoscopic techniques and vertebroplasty (VP). Therefore, the light source should be determined according to each specific situation [64,65,66]. The success of PDT depends not only on the choice of PS and light source, but also on the total light dose and exposure time, as well as other combined treatment strategies.

## 3. Application of PDT in Bone Cancer

### 3.1. Preliminary Studies on the Therapeutic Effect of PDT on Bone Cancer

Possibly due to the poor tissue penetration of the first-generation PSs and the uncertainty about the effect of PDT on normal musculoskeletal tissues, Photofrin, the first PS approved by the FDA, was not studied for bone cancer treatment until the end of the 1980s. Fingar et al. applied PDT for chondrosarcoma in rats using Photofrin II. The release of thromboxane from platelets and endothelial cells in tumors was higher than that in tumor-free tissue, leading to microvascular damage followed by tumor destruction [67]. This vascular damage was also related to changes in tumor interstitial pressure [68]. Meyer et al. demonstrated that bone was very resistant to the effects of PDT while muscle and salivary gland were sensitive to PDT. However, all the normal tissues were noted to heal or regenerate well after PDT injury [69]. Hourigan et al. proved that giant-cell tumor, dedifferentiated chondrosarcoma, and osteosarcoma were susceptible to in vitro PDT and the optimal nontoxic incubation concentration of Photofrin was 3 µg/mL [70]. Subsequently, a large amount of studies on PDT for bone cancer appeared.

### 3.2. PDT Using New Generations of PSs for Bone Cancer

#### 3.2.1. Dextran-Benzoporphyrin Derivatives (BPD)

Recently, numerous in vitro and animal studies on PDT for bone cancer have been performed based on the discovery of hundreds of the second- and third-generation PSs. BPDs for bone cancer therapy are usually used in a liposomal formulation (benzoporphyrin-derivative monoacid ring A, BPD-MA, Visudyne^®^) which was approved by the FDA. BPD-MA was demonstrated to induce long-term chondrosarcoma regression in rats treated with light irradiation 5 min after BPD injection. The timing for light irradiation was related to blood flow stasis which played an important role in PDT-induced tumor destruction [71]. PDT using BPD-MA for primary bone cancer was feasible and effective as reported in the treatment of spontaneous osteosarcomas of the distal radius in dogs [72]. Burch et al. first applied BPD-PDT for bone metastasis. The results showed that BPD-MA selectively accumulated in tumors 3 h post-injection and the MT-1 cells, a human breast cancer cell line, which metastasized to the spine and appendicular bone were eliminated 48 h post-light delivery [73]. Metastatic lesions of MT-1 cells within porcine vertebrae and long bones could also be ablated using BPD-PDT. The average depth of light penetration into trabecular bone was 0.16 ± 0.04 cm while the necrotic/non-necrotic interface extended 0.6 cm. This study demonstrated that the light for BPD-PDT has excellent bone penetration [74]. Akens at al. compared the uptake ratio between BPD-MA and 5-aminolevulinic acid (5-ALA) in spinal metastases in rats. They found 5-ALA did not demonstrate an appreciable uptake difference in tumor-bearing vertebrae compared to spinal cord, while BPD-MA could accumulate specifically in the tumor tissue and reach its highest concentration 15 min after injection. Thus, they speculated that BPD-MA could be used for PDT to treat bone metastasis [75]. Later, they also demonstrated that the safe and effective drug-light dose appeared to be 0.5 mg/kg BPD-MA and less than 50 J light energy for the thoracic spine and 1.0 mg/kg and 75 J for the lumbar spine in rats with bone metastasis of breast cancer [76]. In addition, PDT using BPD-MA was demonstrated to improve vertebral mechanical stability during the treatment of rats with spinal metastasis [77]. Wise-Milestone et al. also found that PDT using BPD-MA promoted new bone formation in non-tumor-bearing vertebrae and suppressed osteoclastic resorption in tumor-bearing vertebrae, leading to a protection of the vertebral structure [78].

#### 3.2.2. Acridine Orange (AO)

AO is a basic dye that can accumulate densely in lysosomes and is specifically taken up by musculoskeletal sarcomas. It is another widely studied PS during last two decades [79,80]. Kusuzaki et al. performed curettage under fluorovisualization and AO-PDT for osteosarcoma elimination in mice. At 2 h after intraperitoneal injection of AO, macroscopic curettage was performed and additional curettage was performed while observing fluorescence of AO bound to residual tumor fragments using a fluorescence stereoscope. Then, the tumor-resected area was irradiated by blue light (466.5 nm) for 10 min to kill the residual cells microscopically. The results showed that local tumor recurrence was significantly inhibited (23%) in the group treated with curettage and PDT, compared to that (80%) in the control group treated with only curettage [81]. At the same time, AO with photoexcitation was demonstrated to have a strong cytocidal effect on multidrug resistance (MDR) mouse osteosarcoma cells [82]. The accumulation of AO in malignant musculoskeletal tumors was possibly related to the pH gradient. The higher the malignancy of the tumor, the greater the pH gradient between the intracellular pH and the extracellular pH or between the intracellular pH and the vacuolar pH are. This acidity of tumors supports AO accumulation [80]. Moreover, different light sources were proved to activate AO and induce cytotoxicity of tumor cells. A study of Ueda et al. showed that strong unfiltered light from a xenon lamp was more effective and feasible than weak filtered blue light for cytocidal effect of osteosarcoma cells using AO-PDT [83]. Satonaka et al. found that the flash wave light (FWL) xenon lamp needed a lower excitation energy and shorter excitation time compared to that of the continuous wave light (CWL) xenon lamp for the cytocidal effect of AO-PDT [84].

#### 3.2.3. Aminolevulinic Acid (ALA)

Due to the poor specificity, there are relatively few reports of ALA used in the treatments of bone cancer [75,76]. However, Dietze et al. confirmed that the intra-articular application of 5-ALA, a precursor of phototoxic molecules, induced a higher protoporphyrin IX (PpIX) accumulation in synovitis tissue compared to non-inflammatory tissue but lower than that in human sarcoma cells (HS 192.T). The results suggested that 5-ALA could be used in the treatment of arthritis [85]. Moreover, ALA-PDT could also result in the death of human osteosarcoma (MG-64) cells in vitro [86]. Coupienne et al. studied the role of receptor-interacting protein 3 (RIP3) and autophagy in PDT for bone cancer using 5-ALA. RIP3 up-regulated the apoptotic and autophagic pathways but the resulting autophagy protected U2OS cells (human osteosarcoma cell line) against PDT-induced cell death. This study indicated that tumor cells themselves could make protective adjustments to the cytotoxicity of the PDT [87].

#### 3.2.4. 5,10,15,20-Tetrakis(meta-hydroxyphenyl)chlorine (mTHPC)

**Figure 1:**
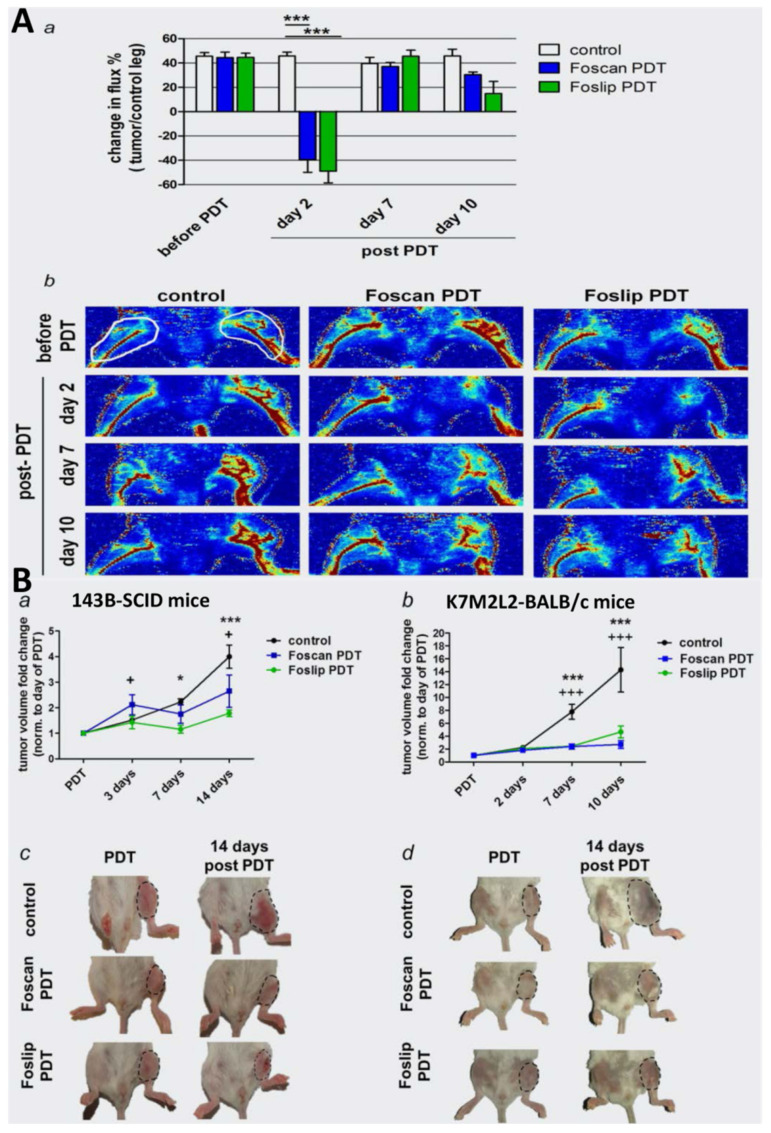
Inhibition of intratibial osteosarcoma growth based on PDT using Foscan or Foslip. (**A**) (**a**) Blood flow in control and tumor-bearing hind limbs was measured with a laser Doppler perfusion imager in the regions of interest (ROI) (as indicated in (**b**)), and the data are presented as percent change in perfusion (flux) of tumor compared to normal tissue, *** *p* < 0.001. (**b**) Images of hind limb perfusion. White circles indicate ROI for laser Doppler image analysis. (**B**) (**a**) Mean fold change in size of intratibial 143B cell line-derived tumors in SCID mice and (**b**) of intratibial K7M2L2 cell line-derived tumors in syngeneic BALB/c mice. + *p* < 0.05, +++ *p* < 0.001, Foscan PDT vs. control; * *p* < 0.05, *** *p* < 0.001, Foslip PDT vs. control. (**c**,**d**) Photographs taken at the end of treatment study. Dotted lines indicate tumor areas. Reproduced from ref. [90] with permission from Wiley Online Library. Copyright (2017) International Journal of Cancer.

The application of mTHPC-mediated PDT for bone cancer emerged in the past decade. mTHPC is currently used in the clinic for palliative treatment of head and neck cancer, traded under the name Foscan^®^ [88]. It could accumulate to higher levels in the high-metastatic 143B cells (human osteosarcoma cell line) than in the parental low-metastatic HOS cells (human osteosarcoma cell line), and the mTHPC-PDT induced caspase-dependent apoptosis [88,89]. Meier et al. encapsulated mTHPC into liposomal structures to improve the drug hydrophilicity. The obtained new PS was labeled Foslip and could be selectively taken up by 143B and mouse K7M2-derived osteosaroma cell line (K7M2L2) (Figure 1). PDT using this new PS eliminated tumors in intratibial human xenografts and syngeneic osteosarcoma mouse models and inhibited lung metastasis [90].

#### 3.2.5. Indocyanine Green (ICG)

ICG is known as a diagnostic drug to check liver function and effective liver blood flow [91,92]. Recently, it has been widely applied in PDT in various fields because of its absorption maximum at ~810 nm [93,94,95]. Funayama et al. investigated the phototoxic effects of ICG on rat mammary adenocarcinoma (CRL-1666) cells and the therapeutic efficacy of ICG-PDT in a rat model of spinal metastasis. ICG-PDT exerted immediate and persistent phototoxic effects on CRL-1666 cells, delayed the deterioration of paralysis, and prolonged the observation period in the spinal metastasis models [96]. Then, they developed a novel nanocarrier consisting of poly(L-lactic acid)-block-poly(sarcosine) labeled with ICG. Under light irradiation, the obtained nanocarrier exhibited tumor selectivity and could be used for the diagnosis and treatment of spinal metastasis [97]. 

#### 3.2.6. Methylene Blue (MB)

MB is a second-generation PS derived from phenothiazine and has been found to kill tumors cells even if they are MDR [98,99]. Matsubara et al. first used MB as a PS for bone cancer. Under the irradiation of light from a 500 W xenon lamp, MB-PDT had a strong cytocidal effect on mouse osteosarcoma (LM8) cells in in vitro experiments, but in vivo studies showed that MB neither selectively accumulated in mouse osteosarcoma tissue nor inhibited tumor growth [100]. However, Guan et al. claimed that PDT using MB and red light from the LED source could remarkably kill rat osteosarcoma-derived UMR106 cells and induce cell apoptosis as well as the collapse of the mitochondrial membrane. They did not perform co-culture of bone tumor cells and normal cells or in vivo experiments [101]. Therefore, the specificity and efficiency of MB-PDT for bone cancer needs further verification. Elfeky et al. used hydroxyapatite nanoparticles to load MB and tested the obtained PS against human osteosarcoma (Saos-2) cells under the light irradiation from a diode array laser. They speculated that the nanocarriers for MB could increase the quantum yield of MB as well as the specificity to cancer cells. The results indicated that the nanocarriers loaded with MB reduced the dose of MB required for effective PDT and this modified PDT had a great cytotoxic effect on macrophage cells which could promote tumor growth [102]. Thus, the nanoformulation is expected to improve the biosafety and efficacy of PSs.

#### 3.2.7. Chlorin e6 (Ce6)

**Figure 2:**
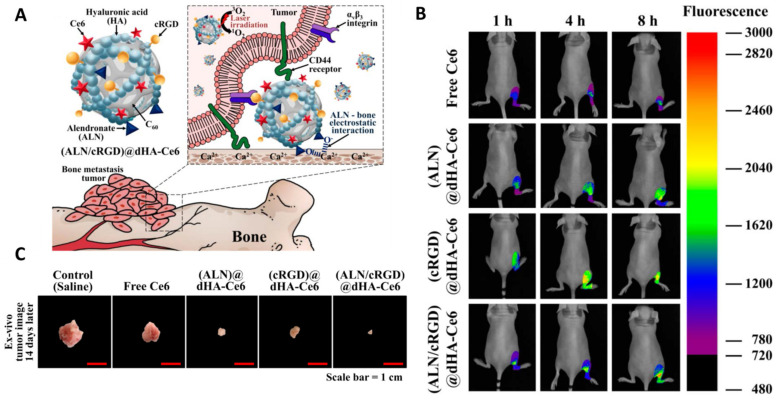
(**A**) Schematic illustration of (ALN/cRGD)@dHA-Ce6. (**B**) In vivo noninvasive photoluminescent tumor imaging of free Ce6 (2.5 mg/kg) or each sample (equivalent Ce6 2.5 mg/kg) intravenously injected into MDA-MB-231 tumor-bearing nude mice. Fluorescent tumor images of the limbs were obtained at 1, 4, and 8 h post-injection. (**C**) Optical images of tumor samples extracted from MDA-MB-231 tumor-bearing nude mice. Reproduced from ref. [106] with permission from MDPI AG. Copyright (2020) Biomedicines.

As the maximum fluorescence excitation and emission wavelength is 403 nm and 669 nm, respectively, and the absorbance peak is at 650 nm, Ce6 can be used not only for in vivo fluorescence imaging of tumors but also for PDT [103,104]. Mohsenian et al. developed Mn-doped zinc sulphide (ZnS) quantum dots loaded with Ce6 for the treatment of chondrosarcoma. Upon exposure of X-rays, the light is generated by the quantum dots and thus activates Ce6. As X-ray irradiation has better tissue penetration, the obtained nanocarriers themselves can serve as an intracellular light source for PS activation which is conducive to eliminating deep tumors [105]. Lee et al. designed hyaluronate dots containing Ce6 with multiligand targeting ability for PDT for bone metastasis. The dots were chemically conjugated with alendronate (ALN, as a specific ligand to bone) and cyclic arginine-glycine-aspartic acid (cRGD, as a specific ligand to tumor integrin αvβ3) for bone and tumor targeting, respectively. The obtained new PS was labeled (ALN/cRGD)@dHA-Ce6. After intravenous injection, these dots sailed to the bone tumor site and were specifically taken up by tumor cells. The multiligand targeting ability was verified by the strong Ce6 fluorescence signal (Figure 2). The bone metastasis in mice caused by human breast carcinoma (MDA-MB-231) cells was inhibited using PDT based on this novel PS [106]. The nanoformulation with targeted recognition technology has the potential for improving the tumor-targeting efficiency of PSs.

#### 3.2.8. Chlorophyll Derivatives

Bacteria are similar to cancer cells as both are highly metabolic and rapidly dividing and can produce lots of porphyrin-derived photosensitizing metabolites [74]. Therefore, some PSs are first used in bactericidal treatment and then found to be also effective for bone cancer, such as chlorophyll derivatives [107,108]. Na-pheophorbide A is a chlorophyll-derived PS with the peak absorption maxima at 410 and 670 nm. PDT with Na-pheophorbide A induced human osteosarcoma (HuO9) cells apoptosis via activation of mitochondrial caspase-9 and -3 pathways [108]. Pd-Bacteriopheophorbide (TOOKAD) is another chlorophyll derivative and its light absorbance is in the NIR region (763 nm), which allows deep tissue penetration [109]. At 70–90 days after PDT, TOOKAD was demonstrated to completely eliminate 50% intratibial metastases caused by implanting human small cell carcinoma of the prostate (WISH-PC2) cells into proximal tibias of mice [110]. As a derivative of chlorophyll, Pyropheophorbide-a methyl ester (MPPa) can be metabolized rapidly and have strong photoelectric sensitivity for PDT. MPPa-PDT was found to induce human osteosarcoma (MG-63) cells apoptosis via the mitochondrial apoptosis pathway and autophagy via the ROS-Jnk signaling pathway. The autophagy could further promote the apoptosis caused by MPPa-PDT [111]. Moreover, MPPa-PDT could block MG-63 cell cycle and inhibit cell migration and invasion. The PDT-induced apoptosis of MG-63 cells was accompanied by the change of cellular endoplasmic reticulum stress (ERS) and related to the Akt/mammalian target of rapamycin (mTOR) pathway [112].

#### 3.2.9. Benzochloroporphyrin Derivatives (BCPDs)

To solve the synthetic problem in the preparation of biologically active BPD-MA and reduce the toxicity to normal tissues, Yao et al. designed and synthesized a novel PS derived from benzochloroporphyrin (BCPD) [113]. After marginal resection of subcutaneous mice tumors caused by the inoculation of a high-metastatic murine osteosarcoma (LM-8) cell line, BCPD-PDT reduced the local recurrence rate and preserved the adjacent critical anatomic structures including muscles, nerves, and vessels [114]. In addition, another report from the same team indicated that BCPD-PDT induced the apoptosis and the cell cycle arrest at the G2M phase of human Ewing sarcoma (TC-71) cells. The tumor volume in mice with Ewing sarcoma in the flank or tibia could be reduced and the function of tumor-bearing limbs was preserved [115].

#### 3.2.10. Other Porphyrin Derivatives

Porphyrin derivatives are the most widely studied PSs, including HpD, BPDs, BCPDs, and so on. Hematoporphyrin monomethyl ether (HMME), a porphyrin-related PS, could be selectively taken up by murine osteosarcoma (LM-8 and K7) cells, while could not be observed in myoblast cells and fibroblast cells. HMME-PDT significantly inhibited subcutaneous osteosarcoma growth in mice via caspase cascade pathways [116]. Hiporfin is a mixture of HpD derivatives and has been approved by the Chinese State Food and Drug Administration for PDT on the oral cavity and the bladder cancers [117]. Sun et al. found hiporfin was as efficient as HMME at a lower concentration, and it could be systemically injected into patients, which is conducive to the PDT for solid tumors. Hiporfin-PDT exhibited cytotoxicity in osteosarcoma in vitro and in vivo by inducing cell apoptosis and necroptosis. However, the resulting cell autophagy played a protective role for tumor cells [118]. Moreover, in order to obtain a PS more active than Photofrin, Serra et al. synthesized 5,15-bis(3-Hydroxyphenyl)porphyrin for PDT [119]. PDT using this new PS reduced tumor size via increasing cell necrosis in murine cranial and vertebral osteosarcomas, which provided a potential platform for surgically inoperable osteosarcoma [120]. Moreover, PpIX is another porphyrin derivative which has been extensively studied in PDT for cancers. The encapsulation of PpIX using silica nanoparticles (SiNPs) improved the efficacy compared to the naked PpIX. Although the encapsulation reduced the PpIX toxicity to tumor cells, the chemicals used for SiNPs synthesis increased the cytotoxicity and thus PDT using PpIX-SiNP significantly inhibited the viability of osteosarcoma cells [121]. In addition to nanoformulation, PSs or PS-carriers can also be internalized by stem cells to further enhance the ability of targeted delivery, as stem cells have the unique ability to home and engraft in tumor stroma. In a report from Duchi et al., Meso-tetrakis(4-sulfonatophenyl)porphyrin (TPPS) was first loaded by fluorescent core-shell poly methyl methacrylate nanoparticles (FNPs), and then the obtained nanocarriers were uploaded by human mesenchymal stem cells (MSCs). Under laser irradiation, the nanocarrier-laden MSCs underwent cell death and released a large amount of ROS to trigger cell death of osteosarcoma cells [122].

#### 3.2.11. Photodynamic Molecular Beacons (PMBs)

As many first- and second-generation PSs are limited by their non-specific uptake in deep tumors such as spinal metastases, PMBs targeting on specific molecules were proposed to localize the active PSs to the tumors [123,124]. PMBs comprise a PS and a quencher moiety which is photodynamically inactive, until transformed into an activated state through cleavage of the linker. Liu et al. synthesize PMBs activated by matrix metalloproteinases (MMPs) and named it PP_MMP_B. It consists of the PS Pyropheophorbide-R and black hole quencher 3, linked by the amino acid sequence GPLGLARK, which is an MMP-cleavable peptide. PP_MMP_B could be specifically taken up and activated by vertebral metastases versus normal tissues [125]. PDT using PP_MMP_B was also demonstrated to ablate metastatic tumors and disrupted the osteolytic cycle, and thus better preserved critical organs in rats with vertebral metastasis [126].

#### 3.2.12. Other New PSs

The development of PSs also draws inspiration from conventional drugs. For example, Aloe-emodin (AE) is an anthraquinone compound extracted from traditional Chinese medicine plants and has antitumor effects. Recently, it was demonstrated to have fluorescence and phototoxicity and could be used in tumor therapy [127,128,129]. Tu et al. found that AE-PDT induced the autophagy and apoptosis of MG-63 cells via the activation of the ROS-JNK signaling pathway [130]. In addition, many third-generation PSs are constructed based on nanoformulation or internalization by cells, which makes them favorable for specific uptake by tumor cells. Lenna et al. developed a PS delivery system using MSCs internalizing FNPs. The PS, tetra-sulfonated aluminum phthalocyanine (AlPcS4), has a strong absorption peak in the NIR region and can retain activity after loading by FNPs [41,131]. FNPs containing AlPcS4 was the uploaded by MSCs. Photoactivation of this PS delivery system decreased the viability of osteosarcoma cells (MG-63, Saos-2, and U-2 OS). The authors claimed that this system has potential for the therapy for MDR tumors and the MSCs-based PDT is conducive to the design of personalized treatment [132].

### 3.3. Combination of PDT and Other Therapies for Bone Cancer

#### 3.3.1. PDT Combined with Chemotherapy

Since most bone cancers involve deep tumors, PDT is often used in combination with chemotherapy, radiotherapy, and immunotherapy to ensure complete ablation and prevent recurrence. The combination of PDT and chemotherapy is widely studied and is called photochemotherapy [133,134]. Systemic bisphosphonates (BP) treatment has been demonstrated to inhibit bone resorption in bone metastasis caused by breast cancer and reduce the fracture chance of involved vertebrae [135]. However, BP is less effective for vertebral tumors beyond a critical size [136]. Therefore, Won et al. proposed a combined treatment of bisphosphonate zoledronic acid (ZA, a derivative of BP) and PDT using BPD-MA. This photochemotherapy not only ablated spinal metastases but also reduced bone loss accompanied by improving the structural integrity of vertebral bones [137]. The combined treatment of ZA and PDT could also reduce the risk of burst fracture and restore the pattern of bone strain to that of healthy vertebrae [138]. The pre-treatment with ZA before PDT reduced the cell viability of MT-1 cells up to 20% compared to PDT alone [139]. Moreover, Heymann et al. combined low-level laser therapy (LLLT) with cisplatin or ZA for bone cancer. They found that the irradiation of low-level laser on Saos-2 cells cultured in medium containing cisplatin or ZA directly raised the cytotoxicity of these two drugs. They speculated that this direct phototoxicity of cisplatin or ZA could be caused by photobiomodulation based on a direct mitochondrial stimulation through LLLT [140]. These results indicate that the combination of PDT and chemotherapeutics drugs synergistically enhances the tumoricidal effect.

Recently, many studies are focusing on the development of nanovehicles which can target PSs and chemotherapeutics drugs on cancer lesions, optimize the shortcomings of drugs, and reduce the side effects of PDT and chemotherapy [141,142,143]. Paclitaxel (PTX) is one of the most effective chemotherapeutics drugs for treating breast, ovarian, lung, and pancreatic cancer [144,145]. To improve its poor water solubility, Martella et al. designed a nanoscale Drug Deliv system consisting of high molecular weight and hydrosoluble keratin, Ce6, and the PTX. PTX and Ce6 acted in an additive manner, and the resulting cytotoxicity to osteosarcoma cells was superior to that of PTX or Ce6 alone. The high specificity and efficiency of this Drug Deliv system is a promising therapeutic strategy for MDR osteosarcomas [146]. Doxorubicin (DOX) is usually used as the first-line therapy for osteosarcoma and doxycycline (DOXY) also has efficient cytotoxicity on various cancer cells. The combination of these two drugs can synergistically induce apoptosis of cancer cells [147,148]. Tong et al. synthesized a prodrug of these two drugs via a thioketal (TK) linkage. The obtained DOX-TK-DOXY was encapsulated into the mesoporous silica nanoparticles (MSNs) followed by modification of Ce6 and ZA. ZA helps the nanocarriers target on osteosarcoma cells and the Ce6 can be activated by laser irradiation and produce ROS. ROS cannot only induce cytotoxicity but also disrupt the TK linkage of the prodrug, leading to synchronous release of both DOX and DOXY. The released DOXY can also promote the production of ROS and thus amplify the release of DOX and DOXY. This nanovehicle with the capacity of bone-targeting, burst release of ROS, and continuous release of chemotherapeutics drugs is a novel therapeutic strategy for bone cancer [149]. Bortezomib (BTZ) is the first clinically approved proteasome inhibitor and can be applied in the treatment of bone cancer. BTZ was found to increase intracellular ROS level which can improve the tumoricidal effects of PDT [150,151]. Huang et al. designed a bone-seeking nanoagent for the treatment of bone metastasis. This nanocarrier comprised ALN (as the bone seeker), Zinc phthalocyanine (ZnPc) (as the PS), and BTZ (as the chemotherapeutics drug and the amplifier of ROS). Tumor volume of bone metastasis in a rat model was cut down by 85% using this photochemotherapy, and the tumoridical effect was related to mitochondrial damage and excessive ERS [152]. In addition, a report from Lu et al. has the similar design concept. In this study, nanoparticles based on graphene oxide (GO) was synthesized. Folic acid was conjugated with GO as a targeted agent for cancer cells, ICG was linked to GO as a PS, and ginsenoside Rg3 was loaded by GO as a chemotherapeutics drug. PDT using the obtained nanocarriers inhibited malignant progression and stemness of osteosarcoma cells [153].

#### 3.3.2. PDT Combined with Immunotherapy

**Figure 3:**
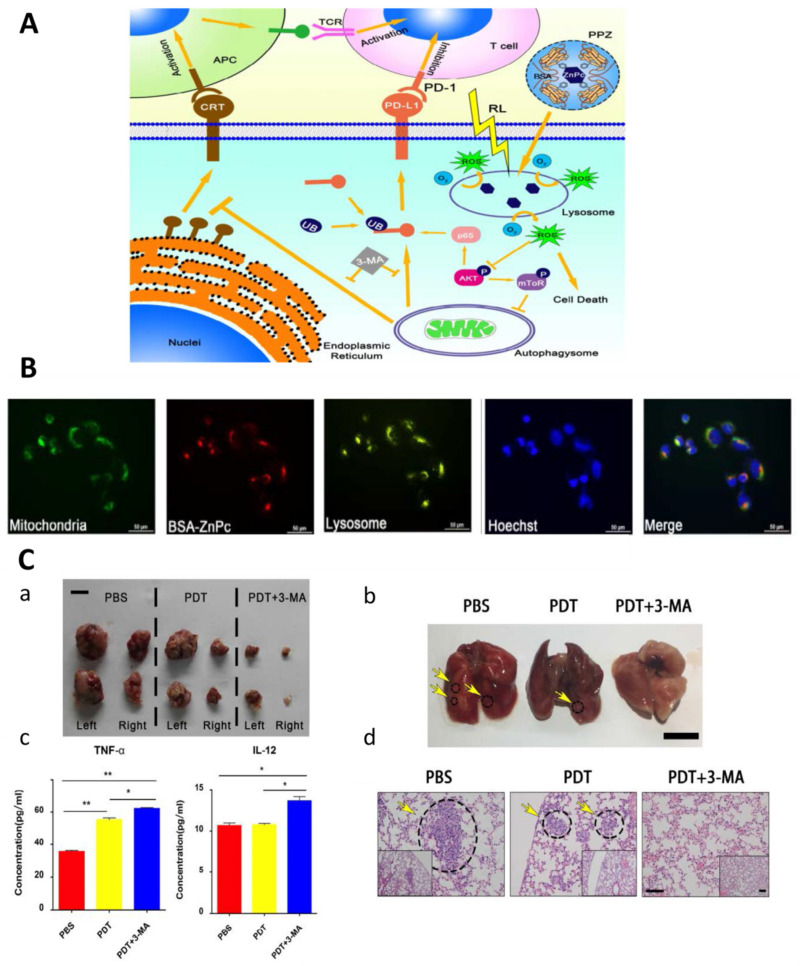
(**A**) Schematic overview of the anti-tumor immune response in osteosarcoma induced by the combination of PDT and 3-MA. (**B**) PDT using BSA-ZnPc induced autophagy in osteosarcoma cell lines. The distribution of mitochondria, lysosomes, and BSA-ZnPc in MNNG/HOS cells after treatment with BSA-ZnPc for 24 h (sacle bar = 50 µm). (**C**) Combined therapy of PDT and 3-MA inhibited tumor growth in a distant metastasis subcutaneous tumor model. (**a**) The tumors located on both flanks were resected and imaged (bar = 5 cm). (**b**,**d**) The images and H&E staining of lungs resected from the tumor-bearing mice (circles represent the metastatic, (**b**): bar = 1 cm, (**d**): left bar = 100 µm, right bar = 200 µm). (**c**) Mice sera were collected 1 day after combination treatment, and the cytokine levels of TNF-α and IL-12 were measured. * *p* < 0.05, ** *p* < 0.01. Reproduced from ref. [166] with permission from Elsevier. Copyright (2019) Biomaterials.

PDT can also induce the immune response to eliminate tumors and prevent recurrence. Due to the complex mechanism involved in this process, there are many target points can be studied for the synergistic treatments of PDT and immunotherapy [154,155]. The combination of PDT and immunotherapy cannot only enhance the anti-tumor immune effects but also reduce the side effects [156,157]. Zhang et al. found that HpD-PDT for osteosarcoma induced necrosis of tumor cells and then inhibited the function of dendritic cells (DCs). However, continuous PDT restored the function of DCs by up-regulating heat shock protein 70 [158]. CpG oligodeoxynuleotide (CPG-ODN), synthesized from unmethylated CpG dinucleotides and a phosphorothioate or chimeric backbone, can stimulate innate immune system via toll-like receptor 9 (TLR9), followed by the activation of DCs and other immune-related cells [159,160,161]. Peritumoral injection of CPG-ODN after PDT using BPD could control both local and systemic tumor spread in mice caused by metastatic breast cancer cells. The therapeutic effect of this combined therapy was improved compared to PDT or CPG-ODN alone [162]. At the same time, Marrache et al. developed a nanoparticle delivery platform based on ZnPc-PDT and CPG-ODN for the treatment of metastatic breast cancer. Polymeric core with gold nanoparticles (AuNPs) were used as a controlled release system for ZnPc and CPG-ODN, and CPG-ODN acted as an immunostimulant to enhance the anti-tumor immunity effect caused by PDT via activating DCs [163]. Moreover, the cytotoxic effects on T cells also play an important role in tumor therapy [164]. When the programmed death ligand-1 (PD-L1)/programmed cell death protein-1 (PD-1) pathway was blocked, PD-1 of tumor cells, an inhibitor of T cell proliferation and cytotoxic effects, was down-regulated followed by significant inhibition of osteosarcoma growth [165,166]. As aforementioned, autophagy may protect tumor cells from the cytotoxicity of PDT [87,118,167]. To suppress autophagy of osteosarcoma cells, 3-MA, an autophagy inhibitor, was applied to enhance the tumoricidal effects of PDT using bovine serum albumin-ZnPc nanoparticles (BSA-ZnPc) (Figure 3). This combination of PDT and immunotherapy inhibited osteosarcoma growth in vitro and in vivo via the inhibition of autophagy and down-regulation of PD-L1 [166].

#### 3.3.3. PDT Combined with Hyperthermia

Hyperthermia has been applied to treat tumors since the 1970s. When the temperature comes to 42 °C or higher, the injury of DNA and plasma membrane and the inhibition of protein synthesis and energy metabolism will occur followed by mitochondrial damage [168,169]. Nomura et al. combined HpD-PDT with hyperthermia (45 °C) to treat osteosarcomas in mice. The tumor growth rate in the heat-only or PDT-only group was significantly lower than that in the group without treatment, and was significantly higher than that in the group treated with PDT and hyperthermia [170]. The combination of ALA-PDT and hyperthermia (43.5 ± 0.5 °C) was also demonstrated to synergistically inhibited the viability of human mandibular osteosarcoma cells. In addition, hyperthermia improved the sensitivity of less sensitive tumor cells to PDT cytotoxicity [171]. These studies on hyperthermia for cancer treatment also inspired the development of PTT.

#### 3.3.4. PDT Combined with Radiotherapy

Radiotherapy with the advantage of palliating pain is recognized as one of the most effective therapies for malignant tumors and is a current standard of treatment of spinal metastasis [172,173]. However, different sensitivities to radiotherapy were found in tumors of different types as well as tumors of the same type but from different individuals [174]. Lo et al. demonstrated that the combination of X-Ray irradiation at 4 Gy and PDT using BPD-MA significantly improved the bone architecture and bone formation of normal vertebrae at a longer-term (6 week) time-point [175]. In addition, this combination maintained the structural integrity of metastatically involved vertebrae in rats while ablating tumors [176]. PDT combined with radiotherapy can provide a potential platform for patients with recurring spinal tumors that cannot be treated by surgery or only radiotherapy [175,176]. 

In addition, the clinical application of PDT for musculoskeletal cancers is often combined with radiotherapy [177]. Synovial sarcoma is one of the most common malignant soft-tissue tumors encountered in children and adolescents with high recurrence rate (~80%) after resection. In addition, it often invades adjacent bones, vessels, and nerves [178,179]. Kusuzaki et al. performed AO-PDT with X-ray irradiation at 5 Gy for six patients with synovial sarcoma after resections. The results showed that the low-dose X-ray also excited AO-like photons. The combination successfully inhibited the recurrence and protected the surrounding normal tissues [180]. Then, they performed PDT or the combined therapy for 4 patients with primary bone cancer and 6 patients with primary malignant soft tissue sarcoma. Among them, 5 patients were treated with AO-PDT and 5 patients were treated with AO-PDT and X-ray irradiation at 5 Gy. After a follow-up of 24–48 months, one of the 5 patients treated with PDT showed local recurrence while there was on recurrence in the 5 patients treated with PDT and radiotherapy [181,182]. Although the number of cases involved is small and the grouping principle is imperfect, these studies still provide a preliminary reference for the clinical application of PDT combined with radiotherapy for bone cancers which are difficult to treat by conventional therapies.

#### 3.3.5. Other Applications of PDT for Clinical Bone Cancer

As chondrosarcoma is radioresistant and often not sensitive to chemotherapy, wide excision surgery is the most common therapy [183,184]. However, when chondrosarcoma occurs in the hyoid bone, many patients choose not to sacrifice the larynx, base of tongue, and the hyoid, and thus surgeries will not be accepted. Therefore, Nhembe et al. chose mTHPC-PDT with the help of bare polished tip laser light delivery fibres for the patient with a 3.4 cm × 2.4 cm chondrosarcoma lesion. At a follow-up after about 20 months, MRI results indicated that the tumor volume decreased accompanied by tissue regeneration and improvement in airway. The residual tumor became smaller and could be seen in the subcutaneous tissue away from the hyoid [185]. In addition to this case, the light source of PDT can also get closer to deep tumors with the help of minimally invasive surgeries [66,77,126,186]. Fisher et al. first applied PDT using verteporfin, a second-generation PS derived from porphyrin, to improve the therapeutic effects of VP or Balloon Kyphoplasty (KP) on patients with pathologic vertebral compression fractures caused by vertebral metastasis. Patients treated with PDT under the light from interstitial diffusing fiber at 50 or 100 J/cm felt pain significantly reduced, and no complications directly attributed to PDT were found. These results suggested that VP or KP combined with PDT is safe and can shorten the hospital stay [187]. Moreover, photochemotherapy based on photochemical internalization (PCI) has been developed for clinical use. PCI is a nano Drug Delivery technology delivering endocytosed macromolecules into the cytoplasm. Upon light activation, PSs located in endocytic vesicles will induce rupture of the endocytic vesicles and release the therapeutic macromolecules into the cytosol. This technology aims to avoid the side effects of PDT and chemotherapy, enhance the efficacy of photochemotherapy, and improve the selectivity of PSs [188,189]. Disulfonated tetraphenyl chlorin (TPCS2a)-based PCI of Bleomycin, a third-generation PS for photochemotherapy, was applied in the treatment of a patient with chondroblastic-osteosarcoma of the jaw. This therapy was demonstrated to have increased selectivity and superior anti-tumor activity compared to PDT only. During the follow-up of three months, continuous tumor shrinkage and death of tumor cells were proven by clinical assessment and histopathology, and no recurrence was identified. Unfortunately, the patient succumbed to cardiorespiratory failure six months after the start of the therapy [190]. Although the first clinical trial of PCI-based photochemotherapy for bone cancer failed to have long-term follow-up, these early follow-up results suggest that this therapy seems to be a feasible clinical therapeutic strategy for bone cancer.

## 4. PTT

PTT for cancer therapy was inspired by magnetic thermal therapy and first reported by Hirsch et al. in 2003. Silica nanoparticles were surrounded by small gold colloid to form gold–silica nanoshell and then modified by polyethylene glycol (PEG) to retain the stability of the nanoshell colloid. After exposure to NIR light (820 nm, 35 W/cm^2^), the human breast carcinoma cells cultured with this obtained PTA lost viability, while cells cultured with only NIR light or PTA kept viability. Therefore, normal tissues which cannot take up a large amount of PTA are safe during PTT [191]. PTA and light source are the two key elements in PTT. When PTAs are irradiated by light with a specific wavelength, the energy from photons will be absorbed by PTAs and PTAs will be activated and collide with surrounding molecules to return to the ground state [192]. Therefore, the increased kinetic energy will be turned into heat. Tumor cells are more sensitive to cytotoxicity caused by heat compared to normal cells. When the local temperature increases to 42 °C or higher, some thermolabile cellular proteins are denatured accompanied by coaggregation with native and aggregative-sensitive proteins, leading to inactivation of downstream pathways, physical alteration of chromatin, inhibition of DNA synthesis and repair, and ultimate cancer cell death [193,194]. PTT for cancer treatment can be performed remotely and applied in combination with conventional therapies, and the intensity, interval, and time of light irradiation can be administrated according each case situation. PTT is a noninvasive, controllable, and targeted strategy to eliminate tumor cells, therefore, it was widely studied for bone cancer therapy in the past decade [29].

Various PTAs and corresponding light sources have been developed and reported since 2003. The light sources with absorption in the NIR region are most commonly used for PTT because of the appropriate tissue penetration capacity and the reduction of photodamage on local normal tissues and cells [41,49,195,196]. PTAs can be divided into four categories, including metal-, carbon-, semiconductor-, and organic molecule-based materials [194,197,198]. Metal-based materials have high photothermal conversion efficiency but the cost is also high and not suitable for widespread clinical use [198,199]. Carbon-based materials have large photothermal conversion area but have poor absorption capacity under NIR light irradiation [200,201,202]. Semiconductor-based materials have high photothermal performance and low cost but further nanoformulation is often required to enhance the specificity and the ability of tumor targeting [197,203]. Most organic molecule-based materials have strong NIR absorption capability, solubility, biocompatibility, and dispersibility, but they also need modification to promote bone regeneration or immunomodulation [204,205]. Studies of these four types of PTAs are constantly progressing, and the main purpose is to improve the photothermal conversion efficiency, solubility, biocompatibility, tumor-targeting capacity, and safety via modification and nanoformulation [206,207,208]. Moreover, recently, PTT is usually combined with other therapies to comprehensively improve the therapeutic effects of bone cancer [209,210,211].

## 5. Application of PTT in Bone Cancer

### 5.1. Metal-Based PTAs

PTT for bone cancer using metal-based PTAs often involves the precious metals including Au and Pt [212,213,214]. Recently, some common metals including Cu, Fe, Bi, and so on, are also widely studied [215,216,217]. These metals are usually applied for PTT via nanoformulation or coating.

#### 5.1.1. Au

**Figure 4:**
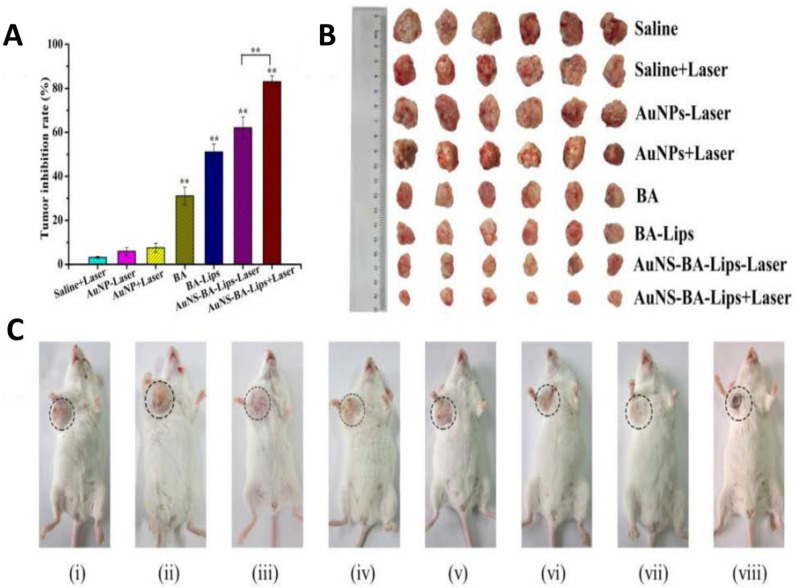
(**A**) The tumor inhibition rates. (**B**) Photographs of tumors after 14 days of treatment. (**C**) Representative photographs of tumor-bearing mice after 14 days of treatment. (i) saline, (ii) saline + Laser, (iii) AuNP-Laser, (iv) AuNP + Laser, (v) BA, (vi) BA-Lips, (vii) AuNS-BALips-Laser, (viii) AuNS-BA-Lips + Laser. The tumor was marked with dashed circles. ** *p* < 0.01. Reproduced from ref. [233] with permission from Elsevier. Copyright (2017) Nanomedicine.

AuNPs have high photothermal conversion efficiency and are one of the most interesting nanomaterials reported in studies on PTT. They are easy to be functionalized via thiol or amine groups for Drug Delivery, and they can generate heat via light irradiation and increase the local temperature to ~43 °C [218,219]. Moreover, the shape and size of them can be altered according to different requirements [220,221,222,223]. Liao et al. used ethacrylated gelatin and methacrylated chondroitin sulfate (CSMA) to encapsulate gold nanorods (GNRs) and nanohydroxyapatite (nHA) to form a hydrogel for bone cancer therapy and bone regeneration. This hydrogel with light irradiation eradicated K7M2wt cells (a mouse bone tumor cell line) and promoted proliferation and osteogenic differentiation of MSCs in vitro. PTT using this hydrogel not only ablated postoperative tumors but also repaired bone defects in a mice model of tibia osteosarcoma [224]. Sun et al. enclosed GNRs in MSNs (Au@MSNs) to form a Drug Delivery platform. ZA was then conjugated to Au@MSNs to provide bone-targeting ability and attenuate tumorigenesis and osteoclastogenesis in bone metastasis. PTT using this composite PTA inhibited tumor growth in vitro and in vivo and relieved bone resorption in vivo [225]. Moreover, CD271 monoclonal antibody was also used as a bone-targeting agent to localize PTAs in osteosarcomas, as CD271 was demonstrated to be overexpressed on the surface of osteosarcoma cancer stem cells [226]. Hollow gold nanospheres (HGNs) were conjugated with SH-PEG-COOH and then CD271 monoclonal antibody was physically absorbed by the obtained PEG-HGNs. The PEG modification was used to increase the stability, reduce cytotoxicity, extend blood circulation time of HGNs, and connect HGNs and CD271 monoclonal antibody [227,228]. This novel PTA could target to osteosarcoma cells and be specifically taken up by the tumor cells. Upon NIR laser irradiation, the cells lost viability [229]. Because AuNPs are conducive to Drug Deliv, PTT using AuNPs is often combined with chemotherapy or immunotherapy [229,230]. Betulinic acid (BA) is a natural anticancer agent against numerous tumor types and has the capacity for local immunoregulation but it is hydrophobic [231,232]. Liu et al. developed gold nanoshell-coated BA liposomes to treat bone cancer. BA was encapsulated into liposomes to increase its solubility, and then coated with AuNPs (AuNS-BA-Lips). The AuNPs nanoshell exerted a prominent PTT effect under the irradiation of light in the NIR region, and the increased temperature triggered BA release (Figure 4). These nanocarriers with dual therapeutic functions inhibited cell viability of 143 B and Hela cells [233]. 

#### 5.1.2. Pt

Unlike Au-based nanomaterials which are non-cytotoxic and have been extensively used in PTT, platinum nanoparticles (PtNPs) are toxic to normal cells [234,235]. Therefore, PTT using PtNPs is required to optimize the size and shape to reduce cytotoxicity [236,237,238,239]. Wang et al. fabricated trifolium-like platinum nanoparticles (TPNs) which showed minimal cytotoxicity to normal cells and could kill cancer cells upon NIR light irradiation. The TPNs inhibited tumor growth and prevented osteolysis in mice with bone metastasis caused by human lung adenocarcinoma (PC9) cells engrafted in the tibias [213]. Yan et al. developed a carboxyl-terminated dendrimer for PtNPs delivery and for targeting to osteolytic lesions in malignant bone tumors. The plentiful carboxyl groups on the dendrimer surface improved the affinity with hydroxyapatite and bone fragments. PtNPs encapsulated by the carboxyl-terminated dendrimer were demonstrated to have minimal cytotoxicity and hematologic toxicity. PTT using the obtained nanocarriers inhibited the tumor growth and tumorassociated osteolysis in mice with bone metastasis caused by injecting MDA-MB-231 cells into tibias [240]. Zhou et al. prepared phytic acid-capped PtNPs with enhanced affinity to hydroxyapatite and osteolytic lesions. These nanocarriers also inhibited the bone tumor growth and the tumor associated-osteolysis in vitro and in vivo upon NIR light irradiation [241].

#### 5.1.3. Cu

Compared with other precious metal-based materials, Cu-based PTAs have the advantages of easy fabrication and low cost. In addition, Cu-based PTAs have better photothermal performance and photostability compared with carbon-based PTAs [242,243,244]. Chang et al. designed copper-doped mesoporous bioactive glass (MBG) for bone cancer. This nanovehicle had both excellent drug loading capacity and photothermal property, and the drug release could be modulated by the photothermal effect. In vitro results showed that PTT using this PTA not only inhibited the tumor cell growth but also induced the formation of apatite mineralization which could promote bone regeneration [245]. Ma et al. developed 3D-printed β-tricalcium phosphate scaffolds coated with MSNs containing Cu for the treatment of residual bone tumors and large bone defects after resection. The composite scaffolds could completely eradiate tumor cells and promote proliferation and osteogenic differentiation of MSCs upon the irradiation of light in the NIR region [246]. Wang et al. prepared platinum-copper alloy nanoparticles modified by aspartate octapeptide, a type of osteotropic peptides, for bone cancer therapy. These nanoparticles could specifically accumulate in bone tumors compared to those without aspartate octapeptide. Under light irradiation, these nanoparticles could not only suppress tumor growth but also reduce the osteoclastic bone destruction [247].

#### 5.1.4. Fe

As Fe can promote the maturation of collagen, and the proliferation and expression of alkaline phosphatase of MSCs, Fe-based materials are also used as PTAs for bone cancer [248,249,250,251]. Liu et al. fabricated 3D-printed bioactive glass-ceramic (BGC) scaffolds containing different metal elements including Cu, Fe, Mn, and Co. Results indicated that Cu-copped scaffolds had the best photothermal performance followed by Fe-copped scaffolds, and PTT using Cu-, Fe-, and Mn-copped scaffolds effectively killed tumor cells in vitro and inhibited tumor growth in vivo. However, only Fe- and Mn-copped scaffolds promoted adhesion and osteogenic differentiation of bone-forming cells. Therefore, Fe-copped scaffolds have more promising potential for PTT-mediated tumor therapy and bone regeneration [217]. In addition, inspired by the previous study, Fe-based materials have the capacities of magnetothermal treatment of osteosarcoma and repairing bone defects [250]. Zhuang et al. fabricated Fe-copped 3D-printed akermanite bioceramic scaffolds with a photo/magnetothermal effect for bone tumor therapy. The simultaneous hyperthermia showed higher heating efficiency compared to single-mode hyperthermia of PTT or magnetothermal therapy, leading to the improved tumoricidal efficiency in vitro. In addition, the composite scaffolds promoted osteogenic differentiation of MSCs compared to scaffolds without Fe [252].

### 5.2. Carbon-Based PTAs

Carbon-based nanomaterials such as graphene-family materials, multi-walled carbon nanotubes (MWCNTs), and carbon dots (CDs) are used as PTAs because of their NIR absorbance, abundant functional groups, and large specific surface area [194,200,201]. The applications of PTT using carbon-based PTAs for bone cancer have been studied over the past decade.

#### 5.2.1. Graphene-Family Materials

Graphene-family materials refer to graphene and its derivatives, including GO, reduced graphene oxide (RGO), and graphene quantum dots (GQDs). Graphene-family materials have a large specific surface area which is conducive to the interaction with other biomolecules, and they have tunable thermal properties to match various demands in biomedicine. They also have good biocompatibility and can promote cell adhesion, proliferation, and differentiation of some types of cells [253,254,255]. Therefore, PTT using graphene-family materials cannot only eliminate bone tumors but also promote bone regeneration. He et al. incorporated graphene nanosheets into polyetheretherketone to form nanofillers. These nanofillers boosted MSCs proliferation in vitro and could reach 45 °C in 150 s upon light irradiation. The obtained nanocomposites have strong potential for PTT and bone regeneration [256]. 

**Figure 5:**
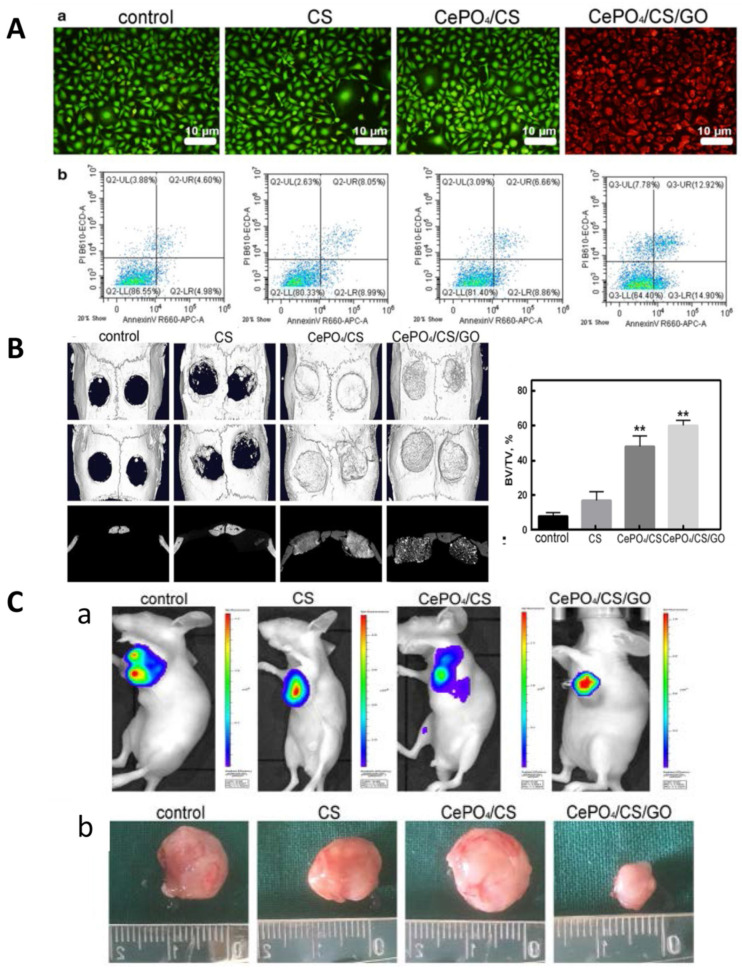
(**A**) (**a**) live-dead cell staining. (**b**) flow cytometry of MDA-MB-231 cells for different groups under NIR laser irradiation for 10 min every day. It was found that the MDA-MB-231 cells in the control, CS and CePO4/CS control groups were live cells, while those in the CePO4/CS/GO group were dead cells (green represents live cells and red represents dead cells). (**B**) Micro-CT images and bone volume/tissue volume (BV/TV) of skulls 3 months after the surgery. ** *p* < 0.01, vs control. (**C**) (**a**) Fluorescence detection on nude mice after NIR laser irradiation and fluorescence intensity of the CePO4/CS/GO group was significantly lower than the blank, CS and CePO4/CS groups. (**b**) Optical picture of tumors in nude mice. Reproduced from ref. [262] with permission from BioMed Central. Copyright (2021) J Nanobiotechnol.

GO is the most widely studied graphene-family PTAs for bone cancer therapy. The functionalization with PEG could enhance the dispersion and stability of GO [257,258]. After PEG-GO nanosheets (40 µg/mL) were taken up by pre-osteoblasts (MC3T3-E1 cells), the cells retained normal ALP levels and matrix mineralization. These nanomaterials are promising PTAs for the treatment of bone cancer [259]. Guo et al. developed a multifunctional scaffold consisting of porous polyurethane (PU) substrate with GO nanosheet/chitosan (CS) hybrid coatings via layer-by-layer assembly process. The GO-based coating can load with a variety of drugs, such as MB, silver nanoparticles, and fluorescein sodium for multiple purposes. The drug release can be controlled by local pH value and the photothermal effects can be activated upon light irradiation [260]. Xu et al. introduced GO nanosheets into ricalcium silicate particles via co-precipitation to fabricate dual functional bone cement. The photothermal performance of this cement can be regulated by the laser power and the GO content. This cement could not only ablate bone tumor cells but also promote cell proliferation and enhance the ALP activity of MC3T3-E1 cells [261]. Ge et al. prepared multifunctional scaffolds that comprised GO nanoparticles, hydrated CePO4 nanorods, and CS. Under NIR laser irradiation, the GO component can exert photothermal effect to kill tumor cells. The hydrated CePO4 nanorods could induce M2 polarization of macrophages which secretes vascular endothelial growth factor (VEGF) and arginase-1 (Arg-1), and activate the BMP-2/Smad signaling pathway, promoting bone regeneration (Figure 5). This composite scaffold is a promising candidate for angiogenesis and osteogenesis after bone tumor resection [262].

In addition to GO, rGO and GQDs are also applied for PTT. Li et al. developed a composite scaffold consisting of nHA and rGO sheets via self-assembly. The scaffolds killed 92% of MG-63 cells and inhibited tumor growth under laser irradiation at 808 nm for 20 min. At the same time, the scaffolds promoted adhesion, proliferation, and osteogenic differentiation of MSCs in vitro and enhanced bone regeneration in rats with calvaria defects [263]. Liu et al. adjusted the absorbance of GQDs to 1070 nm in the NIR-II region to make the light have stronger tissue penetration. GQDs were treated with phenol by tuning the decomposition of hydrogen peroxide under a high magnetic field of 9T, the obtained nanomaterials were labeled 9T-GQDs. 9T-GQDs had tunable fluorescence and high photothermal conversion efficacy (33.45%). Both in vitro and in vivo results showed that 9T-GQDs could ablate tumor cells and inhibit tumor growth under laser irradiation in the NIR-II region. In addition, 9T-GQDs exhibited obviously NIR imaging of tumors in living mice, suggesting the probability of 9T-GQDs for imaging guided PTT [264].

#### 5.2.2. MWCNTs

MWCNTs are a class of nanotubes, and can absorb more NIR irradiation and load with more drugs due to the larger surface area compared to conventional single-walled carbon nanotubes (SWCNTs) [265,266]. Moreover, more absorption of NIR irradiation can reduce the side effects of light irradiation. The superior capacities of photothermal conversion efficiency and Drug Delivery make MWCNTs more appropriate for PTAs and for PTT combined with chemotherapy or immunotherapy [267,268,269]. PEGylated MWCNTs were fabricated via sonicating MWCNTs with DSPE-PEG 2000. The hydrophobic surface of MWCNTs is transformed into hydrophilic via PEG modification. MWCNTs injection with NIR laser irradiation remarkably inhibited tumor growth of bone metastases in mice tibias caused by the injection of murine breast cancer (EMT6) cells, while MWCNTs or light irradiation alone exhibited no cytotoxicity to breast carcinoma cells in vitro [270]. Saber-Samandari et al. entrapped carboxyl-functionalized MWCNTs and Fe_3_O_4_ into scaffolds consisting of gelatin and akermanite. The addition of MWCNTs and Fe_3_O_4_ endowed the scaffolds with the capacities of photothermal conversion and improved the mechanical properties of the scaffolds. The obtained scaffolds could rapidly increase to 43 °C under NIR laser irradiation for 10 s. This composite scaffold is potential for ablating residual tumor cells and promoting bone regeneration after resection [271]. 

#### 5.2.3. Other Carbon-Based PTAs

Unlike many carbon-based nanomaterials, CDs not only exhibit photothermal effects but also have water solubility and low cytotoxicity, and are cost-effective [272,273,274]. Lu et al. developed CD doped chitosan/nHA scaffolds which remarkably reduced osteosarcoma cells in vitro and inhibited tumor growth in vivo upon NIR laser irradiation. The scaffolds could also eliminate bacteria (S. aureus and E. coli) under light irradiation. In addition, CD doped scaffolds promoted adhesion and osteogenesis of MSCs in vitro and improved the bone formation at 4 weeks after implantation compared to pure chitosan/nHA scaffolds. Therefore, the application of CDs enhanced the osteogenesis-related capacity of scaffolds and endowed the scaffolds with potential for PTT to treat bone tumors and infections [275]. Carbon aerogel (CA) with 3D open networks is another carbon-based material for PTT. Due to the large surface area, ultralow density, and high porosity, it is suitable for the coating of materials [276,277]. Dong et al. designed a multifunctional beta-tricalcium phosphate bioceramic platform coated with CA. CA coating not only exhibited photothermal effects on ablating osteosarcoma but also promoted bone regeneration in rats via a fibronectin-mediated signaling pathway [278].

### 5.3. Semiconductor-Based PTAs

Semiconductor-based materials are metal and non-metallic compounds which can reduce the consumption and cytotoxicity of metal-based materials and improve the photothermal conversion efficiency of non-metallic materials. Due to these excellent characteristics, recently, they are in the most exciting part of the studies on PTAs [194,279,280].

#### 5.3.1. MXene Nanaosheets

**Figure 6:**
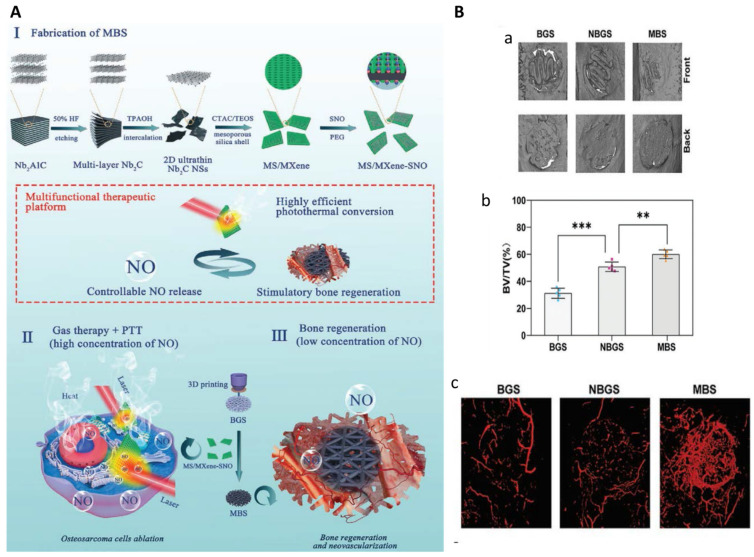
(**A**) Schematic illustration of the multifunctional therapeutic platform. (**B**) In vivo combinatory performance of bone formation and neovascularization. (**a**,**c**) 3D reconstruction of cranial defects and vessels at week 16 after implantation. (**b**) Quantitative fundamental parameters of bone volume/tissue volume (BV/TV) in newborn osseous tissue. ** *p* < 0.01 and *** *p* < 0.001. Reproduced from ref. [291] with permission from Wiley Online Library. Copyright (2020) Small.

In MXene nanaosheets, ‘M’ refers to transition metal atoms, ‘X’ means carbon or nitrogen, and ‘ene’ represents ultrathin 2D structure such as graphene [281]. As MXene nanosheets combine the advantages of metallic materials and non-metallic materials, they have been widely used in biomedicine including biosensing, fluorescent imaging, and PTT [282,283,284,285]. Pan et al. explored the PTT effects of 3D-printed bioactive glass (BG) scaffolds containing titanium carbide (Ti_3_C_2_) nanosheets on the treatment for osteosarcoma. The incorporation of Ti_3_C_2_ MXenes endowed the composite scaffolds with high photothermal conversion efficiency, leading to complete tumor eradication in mice with xenografts of Saos-2 cells. The composite scaffolds could also accelerate bone regeneration after implantation [286]. Yang et al. developed 3D-printed BG scaffolds (BGS) incorporated with S-nitrosothiol-grafted mesoporous silica containing niobium carbide (Nb_2_C) nanosheets (MBS) for the treatment of bone cancer (Figure 6). Upon NIR laser irradiation, photothermal conversion could be achieved via Nb_2_C MXenes and nitric oxide (NO) release could be triggered and controlled. Tumor ablation was strengthened by the combination of MXene-mediated PTT and NO release, as NO at high concentrations could induce DNA damage and inhibition of DNA repair [287,288]. The tunable NO release could also promote vascularization and osteogenesis [289,290]. Therefore, this composite scaffold has the potential for a multifunctional therapeutic platform for osteosarcoma therapy, vascularization, and bone regeneration [291]. Recently, Yin et al. develop implants with multiple functions which comprised Ti3C2 MXenes loading with tobramycin (an antibacterial drug), gelatin methacrylate (GelMA) hydrogels, and bioinert sulfonated polyetheretherketone (PEEK). PEEK substrates was first coated with polydopamine (PDA) to enhance the adhesion of the surface, and tobramycin-laden MXenes was then bonded to PEEK followed by GelMA coating. The combination of MXenes and PDA endowed the composites with synergistic photothermal effects, and the GelMA coating promoted bone regeneration. The results showed that the obtained composite implants exhibited superior cytocompatibility, antibacterial effect, PTT-mediated anti-tumor effects, and the capacity of promoting osteogenesis [292].

#### 5.3.2. Oxide Semiconductor-Based Materials

Biocompatible conductive oxide semiconductors which have photothermal convertible efficiencies and photostability can be used as PTAs [293,294]. SrFe12O19 nanoparticles were synthesized by Lu et al. MBG/CS porous scaffolds containing SrFe12O19 nanoparticles were demonstrated to trigger osteosarcoma apoptosis and ablation upon NIR laser irradiation. The composite scaffolds also promoted bone regeneration via activating BMP-2/Smad/Runx2 signaling pathway [295]. Then DOX was loaded by this composite scaffold. DOX could be rapidly released from the scaffold with the light irradiation, and the resulting chemotherapy synergistically enhanced the anti-tumor effect of PTT [296]. Jie et al. developed oxygen vacancy-rich tungsten bronze nanoparticles (Na_x_WO_3_) via a pyrogenic decomposition process for PTT. These nanoparticles could increase their temperature from 25.8 °C to 41.8 °C in 5 m under the irradiation of 980 nm laser. PTT using these nanoparticles could both eliminate the subcutaneous and intratibial tumors caused by the injection of murine breast cancer (4T1) cells [297]. In addition, the hydrogenated TiO_2_ coating with hierarchical micro/nano-topographies was fabricated by induction suspension plasma spraying. This coating exhibited excellent and controllable photothermal effect on inhibiting tumor growth under NIR laser irradiation in vitro and in vivo. The hierarchical surface of the coating promoted adhesion, proliferation, and osteogenic differentiation of rat MSCs. This coating is potential for bone cancer therapy and bone regeneration [298].

#### 5.3.3. Metal-Organic Frameworks

Metal–organic frameworks (MOFs), 2D nanosheets constructed by metal ions or clusters and organic ligands, have also been used as PTAs [299,300]. The structure and function can be precisely tuned by altering the metal or organic component [301]. Qu et al. designed a multifunctional injectable MOF consisting of cobalt coordinated tetrakis(4-carboxyphenyl)porphyrin (Co-TCPP). Then calcium phosphate cement (CPC) was modified by this MOF for minimally invasive treatment of neoplastic bone defects. The addition of MOF endowed CPC with the improved compressive strength, shortened setting time, and excellent photothermal performance. The composite cement not only ablated tumors in vitro and in vivo but also promoted osteogenesis and angiogenesis in vivo [302]. In addition, Dang et al. prepared copper coordinated tetrakis(4-carboxyphenyl)porphyrin (Cu-TCPP) as a coating for 3D-printed β-tricalcium phosphate scaffolds. The composite scaffolds could significantly kill osteosarcoma cells in vitro and ablate the subcutaneous bone tumor tissues in vivo under NIR light irradiation. In addition, they can also supported the attachment of MSCs and human umbilical vein endothelial cells (HUVECs), and promoted osteogenesis and angiogenesis in rabbits with femoral defects [303]. 

#### 5.3.4. Other Semiconductor-Based Materials

To endow the bioceramics with PTT effects for bone cancer therapy, Wang et al. incorporated nano PTAs into the bioceramics. They synthesized a series of bioceramics via magnesium thermal reduction based on phosphate-based (e.g., Ca_3_(PO_4_)_2_, Ca_5_(PO_4_)_3_(OH)) and silicate-based ones (e.g., CaSiO_3_, MgSiO_3_), and the color of these bioceramics changed from white to black, so they called the obtained bioceramics black ceramics. Due to the oxygen vacancies and structural defects within the crystals, the black ceramics exhibited excellent photothermal effect under NIR laser irradiation. These black ceramics had controlled degradability matching with the bone regeneration rate and promoted bone repair. In addition, upon light irradiation, they exhibited anti-cancer effects on both skin and bone tumors [304]. Ti-based ceramics with good biocompatibility are low-cost semimetal material and widely used in surgical tools, bone repair, and PTT [305,306]. TiN is one of the Ti-based ceramics and was used as a coating for tricalcium phosphate scaffolds in a report from Dang et al. The coated scaffolds also loaded with DOX so as to achieve synergistic tumoricidal effects of PTT and chemotherapy for bone cancer therapy. The in vitro and in vivo results indicated that this composite scaffold effectively eradicated tumors upon light irradiation, suggesting that this composite could be used as implanting material for bone defects after surgical interventions [307]. Cu-based chalcogenides are another widely used PTAs due to the low cost, easy fabrication, tunable size and composition, high photothermal conversion efficiency, and good photostability [242,243,308,309]. Dang et al. fabricated 3D-printed BGS functionalized by CuFeSe_2_ nanocrystals. The scaffold could effectively kill Saos-2 cells in vitro and significantly inhibit bone tumor growth in vivo under light irradiation. At the same time, the scaffolds promoted osteogenic differentiation of MSCs and facilitated bone formation in the bone defects [310].

### 5.4. Organic Molecule-Based PTAs

Organic molecule-based PTAs have aroused widespread interest among researchers. They are characterized by water solubility, good biocompatibility, and easy bioconjugation [204,311]. They mainly include organic NIR dyes and conductive polymers [312,313]. 

#### 5.4.1. Organic NIR Dyes

Fluorescence imaging for bone cancer therapy based on NIR dyes has the advantages of visible delivery and therapy [314,315,316]. ICG is a medical imaging and diagnosis NIR dye approved by FDA for clinical use [317,318]. As mentioned above, it can be used not only for PDT but also for PTT. MSCs, nanoparticles, and hydrogels are often used as the carriers of ICG to target to and then accumulate in tumors [319,320,321]. Jiang et al. designed bone-targeting nanoparticles with photothermal effects for bone cancer treatment. They conjugated superparamagnetic Fe_3_O_4_ nanoparticles with ZA followed by ICG modification. ZA acted as a bone-targeting factor, while Fe_3_O_4_ and ICG were employed as PTAs to enhance the PTT effect. ICG could also provide the capacity of real-time fluorescence monitoring during the treatment. These nanoparticles could rapidly and accurately located in the medullary cavity of the mice tibia, and then ablated the tibial metastasis of breast cancer cells [322].

#### 5.4.2. Conductive Polymers

Conductive polymers are promising for clinical PTAs as they are cost-efficient and their structures can be precisely controlled [204,323,324]. They are usually used as coatings or crosslinkers to modify scaffolds or nanoparticles, leading to materials with multifunction [324,325]. PDA is the most widely used conductive polymer in PTT [326,327,328]. It is the main component of melanin and has good biocompatibility, low toxicity, and biodegradability. Its intense absorption is in the NIR region (700–1100 nm) and its photothermal conversion efficiency is as high as 40% [326,329,330]. Ma et al. coated 3D-printed bioceramic scaffolds with PDA for bone cancer therapy. The scaffold could support attachment, proliferation, and osteogenesis of MSCs. After light irradiation, the scaffold could induce cell death of Saos-2 and MDA-MB-231 cells in vitro and inhibit the growth of subcutaneous tumor [325]. Wang et al. developed ALN-conjugated PDA nanoparticles loaded with SN38 (a chemotherapeutic drug) for bone-targeting chemo-photothermal therapy for bone cancer. ALN could enhance the affinity to hydroxyapatite in bones and the release of SN38 could be triggered by NIR laser irradiation. PTT using these bone-targeting nanoparticles suppressed the growth of bone tumors and reduced the osteolysis [331]. Luo et al. fabricated an injectable hydrogel consisting of oxidized sodium alginate and chitosan, and the hydrogel contained cisplatin for chemotherapy and PDA-decorated nHA for PTT and bone repair. Under light irradiation, this hydrogel ablated 4T1 cells in vitro and suppressed tumor growth in vivo. In addition, the hydrogel could also promote adhesion, proliferation, and ostegenic differentiation of MSCs in vitro, and enhance bone regeneration in vivo [332]. MSCs can be used as a Drug Delivery system to target on tumor cells because of the hypo-immunogenicity and migration capacity; however, MSCs may promote the progression and metastasis of tumor cells [333,334]. Therefore, stem cell membrane which also has bone-targeting ability and is safer than MSC, was chosen to be the delivery system for PDA nanoparticles to treat bone cancer. Stem cell membrane-camouflaged PDA nanoparticles loading with SN38 exhibited lower nonspecific macrophage uptake, longer retention in blood, and more effective accumulation in tumors than that shown by nanoparticles without stem cell membrane. These obtained nanoparticles showed synergistic anti-tumor effects of PTT and chemotherapy on MG63 cells [334]. Recently, Yao et al. prepared 3D-printed scaffolds based on hydroxyapatite, PDA, and carboxymethyl CS for bone cancer therapy. The incorporation of PDA remarkably enhanced the rheological properties of the slurry for molding, mechanical properties, surface relative potential, and water absorption of composite scaffolds, and also endowed the scaffolds with pthotothermal capacity. Under light irradiation, the scaffolds could not only inhibit tumor growth but also promote osteogenic differentiation of MSCs [335].

### 5.5. Combination of PTT and PDT

**Figure 7:**
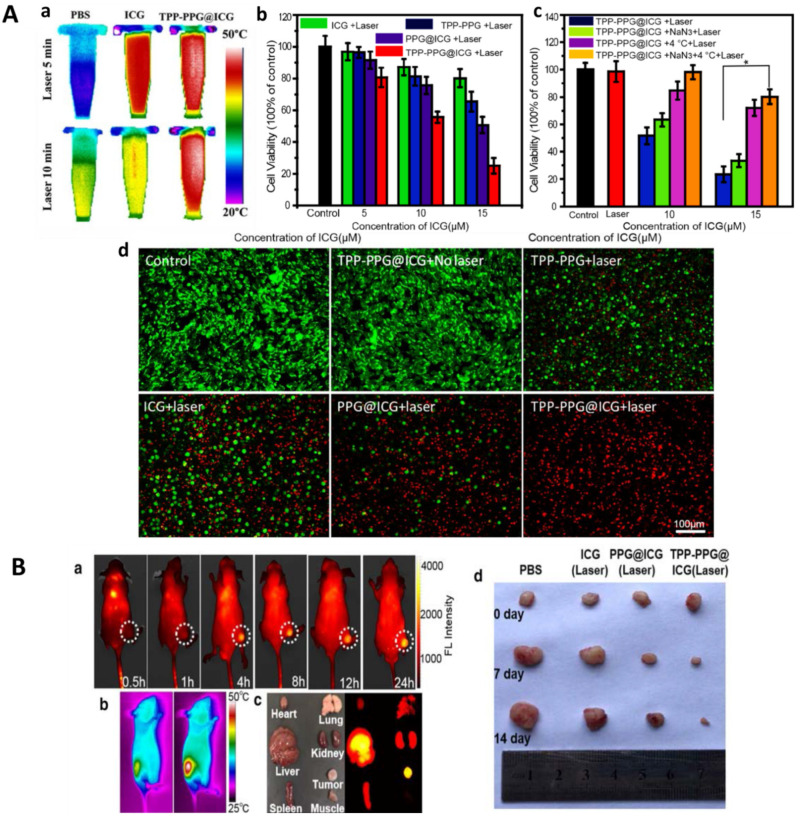
(**A**) (**a**) Thermal images during a 5-min irradiation (upper row) or a 10-min irradiation (bottom row). (**b**–**d**) Synergistic in vitro PT effects of TPP-PPG@ICG and laser irradiation on MG63/Dox cells. (**b**) CCK-8 viability assay at 24 h after laser irradiation. (**c**) CCK-8 viability assays with MG63/Dox cells exposed to TPP-PPG@ICG at 24 h post-laser irradiation at 808 nm, under conditions that inhibited PDT or PTT. (**d**) Images of calcein AM + PI co-stained cells. * *p* < 0.01. (**B**) In vivo synergistic PT for MG63/Dox tumor xenografts. (**a**) NIR fluorescence imaging of tumor xenografts and (**c**) the dissected organs from the tumor-bearing mice. (**b**) IR thermal imaging of tumor xenografts. (**d**) Photographs of representative tumors resected from different groups. Reproduced from ref. [343] with permission from BioMed Central. Copyright (2021) J Nanobiotechnol.

Since the design of PSs and PTAs is transformed to nanoformulation, and the optimal light source for PDT and PTT is in the NIR region, many novel nanocarriers, which can play the roles of both PS and PTA, were reported recently [336,337,338,339]. The resulting enhanced PT using these nanocarriers is called synergistic PT. In addition, these nanocarriers can also load with chemotherapeutic drugs and immunoregulatory drugs to improve the anti-tumor efficacy in multiple aspects. Cheng et al. synthesized AgBiS_2_ nanoparticles for the synergistic PT for bone cancer. These nanoparticles could convert light into heat with a high photothermal conversion efficiency of 36.51% and remarkably increase the generation of intracellular ROS under NIR laser irradiation. The synergistic PT effectively inhibited the growth of malignant osteosarcomas in vivo and also reduced the viability of S. aureus in vitro [340]. Moreover, as ICG exhibits both PDT and PTT effects under light irradiation, ICG-based nanovehicles can be used for the synergistic PT [341,342]. Zeng et al. developed ICG-laden GO nanosheets modified by (4-carboxybutyl) triphenyl phosphonium bromide (TPP, a mitochondria-targeting ligand), for osteosarcoma therapy, and the obtained nanocarriers were labeled TPP-PPG@ICG. The synergistic PT effects of PDT and PTT were confirmed by the detection of intracellular ROS and thermal imaging, respectively (Figure 7). These mitochondria-targeting nanosheets could, in particular, accumulate in tumor cells and significantly eradicate MDR osteosarcomas under light irradiation [343].

## 6. Conclusions and Outlooks

As some bone cancer cells may remain or recur in the local area after tumor resection, some are highly resistant to chemotherapy, and some are insensitive to radiotherapy, there are multiple undesirable results following bone cancer therapy, such as motor dysfunction, neurological symptoms, reduced quality of life, and mental and economic burdens. PT including PDT and PTT, has the advantages of minimally invasive, highly efficient and selective, and easy to combine with other treatments. Therefore, PT is recognized as a new generation of effective treatment for bone cancer. The most used light source in PT is the light with absorbance in the NIR region, which possesses sufficient tissue penetration and minor side effects, and can induce the generation of intracellular ROS or photothermal conversion to ablate tumor cells. Studies on PDT for bone cancer are mainly focused on the development and optimization of PSs, in order to improve the safety and efficiency of the second- or third-generation PSs. Nanoformulation is the main trend in the development of PSs which can endow PSs with bone- or tumor-targeting capacity, the ability of loading chemotherapeutic or immunotherapeutic drugs, and enhanced biocompatibility and residence time. For PTT, semiconductor-based and organic molecule-based PTAs are the most interesting PTAs in recent years due to the low biotoxicity and cost and high photothermal conversion efficiency. Designs of PTAs often take into account the capacity of promoting bone regeneration which can accelerate bone repair in the neoplastic bone defects, as well as the drug loading ability to combine with chemotherapy and immunotherapy. In addition, nanocarriers based on metal nanoparticles or organic NIR dyes exhibit both PDT and PTT effects, and the resulting synergistic PT has stronger tumoricidal effects while the side effects are not improved. Moreover, some researchers are focusing on the specific mechanisms of PT effects on tumor therapy and they want to further improve the effects via altering the expression of involved molecules in corresponding signaling pathways [344]. Recently, computerized medical imaging has also been employed for the diagnosis, planning, and real-time monitoring during PT [345].

However, there are also some crucial challenges or opportunities for further clinical applications of PT. First, the PDT efficiency and side effects depend on the time, intensity, and interval of light irradiation, as well as the amount of PSs. Therefore, guidelines for the clinical use of PDT are necessary. When PDT combined with minimally invasive techniques such as endoscopy is used for deep bone cancer, the clinical protocol can be customized according to existing ones for other superficial tumors. Secondly, unlike studies on PDT, studies on PTT mainly focus on the design and development of PTAs, but the clinical experiments in PTT are rarely reported. The progress of PTT in clinical application lags far behind that of PDT. Thirdly, the long-term metabolism and biocompatibility of the nanoscale PSs and PTAs, and the tumor-targeting capacity and specificity of PSs and PTAs for various cancers, are required further studies. Fourthly, pre-clinical and clinical experiments in real-time monitoring for local immune response and situations of surrounding normal tissues are also needed. Finally, although the synergistic PT and PT combined with other conventional treatments are the most interesting area among studies, the necessity, economic benefits, safety, and efficacy of these combined therapies require detailed discussion depending on each individual. In summary, PT for bone cancer has developed rapidly in recent years, and we strongly believe that PT has great prospects in tumor therapy. We hope this review can provide valuable information and insights for future studies on PT.

## Data Availability

Not applicable.
